# Psychological evaluation in bariatric surgery candidates: the absence of eating disorders does not always mean absence of risk

**DOI:** 10.1192/j.eurpsy.2026.11373

**Published:** 2026-07-31

**Authors:** C. Garcia Cerdan, M. Ligero Argudo, I. M. Peso Navarro, A. I. Mitadiel Velasco, C. Munaiz Cossio, M. Covacho González, M. Benavides Madariaga, I. Sánchez Díez

**Affiliations:** 1 Psychiatry; 2 Psychology, University Clinical Hospital, Salamanca, Salamanca, Spain

## Abstract

**Introduction:**

Severe obesity is often accompanied by life trajectories marked by dieting, restriction, and stigma. Although many bariatric surgery candidates do not meet diagnostic criteria for eating disorders (EDs), their histories may reveal psychological vulnerability and difficulties in their relationship with food. Psychological evaluation is essential to detect these nuances and guide prognosis.

**Objectives:**

To describe two cases of women with morbid obesity undergoing bariatric surgery assessment, without an ED diagnosis on psychometric testing, but with life histories shaped by restrictive dieting since childhood, bullying, and frustration related to weight loss and regain.

**Methods:**

Comparative analysis of two psychological assessments conducted in bariatric surgery outpatient clinics, including structured clinical interview, psychometric tests (BDI, Goldberg, BES, EDI-2, Salamanca Questionnaire), and review of life history and eating habits.

**Results:**

In both cases, patients were considered suitable for surgery, with no evidence of current ED symptoms.

- **Case 1:** A 35-year-old woman with obesity since childhood and a marked history of restrictive dieting starting at age 6 (“they sent me to school with an apple”). She reported deprivation, fatigue, and limited nutritional education as contributing to weight regain after significant weight loss. No binge or compensatory behaviors were identified.
- **Case 2:** A 41-year-old woman with a history of bullying in adolescence due to her weight, reporting binge eating episodes during that period. She described a long history of medical and non-medical diets, as well as a previous gastrectomy with subsequent weight loss and regain. Currently, she does not report disordered eating behaviors.
- Both patients reported good quality of life and strong motivation to improve functionality, although they presented physical limitations and emotional vulnerability related to weight and the chronic experience of dieting.

**Conclusions:**

These cases illustrate that the absence of a formal ED diagnosis does not equate to absence of risk in bariatric surgery candidates. Histories of restrictive dieting since childhood, weight-related stigma, and frustration from repeated weight loss and regain are psychological vulnerabilities that may influence postsurgical outcomes. Psychological evaluation can be an improvement beyond psychometric screening, allowing hidden risks to be identified and informing recommendations for comprehensive care.

**Disclosure of Interest:**

None Declared

